# Molecular cloning, sequencing and tissue expression of vasotocin and isotocin precursor genes from Ostariophysian catfishes: phylogeny and evolutionary considerations in teleosts

**DOI:** 10.3389/fnins.2015.00166

**Published:** 2015-05-15

**Authors:** Putul Banerjee, Radha Chaube, Keerikkattil P. Joy

**Affiliations:** ^1^Department of Zoology, Centre of Advanced Study, Banaras Hindu UniversityVaranasi, India; ^2^Zoology Department, Mahila Mahavidhylaya, Banaras Hindu UniversityVaranasi, India

**Keywords:** catfish, cloning, phylogeny, vasotocin-isotocin precursors, synteny analysis

## Abstract

Basic and neutral neurohypophyseal (NH) nonapeptides have evolved from vasotocin (VT) by a gene duplication at the base of the gnathostome lineage. In teleosts, VT and IT are the basic and neutral peptides, respectively. In the present study, VT and IT precursor genes of *Heteropneustes fossilis* and *Clarias batrachus* (Siluriformes, Ostariophysi) were cloned and sequenced. The channel catfish *Icatalurus punctatus* NH precursor sequences were obtained from EST database. The catfish NH sequences were used along with the available Acanthopterygii and other vertebrate NH precursor sequences to draw phylogenetic inference on the evolutionary history of the teleost NH peptides. Synteny analysis of the NH gene loci in various teleost species was done to complement the phylogenetic analysis. In *H. fossilis*, the NH transcripts were also sequenced from the ovary. The cloned genes and the deduced precursor proteins showed conserved characteristics of the NH nonapeptide precursors. The genes are expressed in brain and ovary (follicular envelope) of *H. fossilis* with higher transcript abundance in the brain. The addition of the catfish sequences in the phylogenetic analysis revealed that the VT and IT precursors of the species-rich superorders of teleosts have a distinct phylogenetic history with the Acanthopterygii VT and IT precursors sharing a less evolutionary distance and the Ostariophysi VT and IT having a greater evolutionary distance. The genomic location of VT and IT precursors, and synteny analysis of the NH loci lend support to the phylogenetic inference and suggest a footprint of fish- specific whole genome duplication (3R) and subsequent diploidization in the NH loci. The VT and IT precursor genes are most likely lineage-specific paralogs resulting from differential losses of the 3R NH paralogs in the two superorders. The independent yet consistent retention of VT and IT in the two superorders might be directed by a stringent ligand-receptor selectivity.

## Introduction

The neurohypophyseal (NH) nonapeptides are an evolutionarily old family which is believed to have originated much before the vertebrates, as one of the first neurotransmitters in the archetype metazoan nervous system (Grimmelikhuijzen et al., 2002). Starting from the most primitive metazoan- the cnidarians, the presence of nonapeptide homologs has been documented in several nonvertebrate phyla (Grimmelikhuijzen et al., 1982; Cruz et al., 1987; Proux et al., 1987; Satake et al., 1999; Takuwa-Kwodo et al., 2003; Kawada et al., 2008; Stafflinger et al., 2008). In vertebrates, studies are relatively extensive and the nonapeptide precursor genes have been cloned from several vertebrate groups: cyclostomes (Lane et al., 1988; Suzuki et al., 1995; Gwee et al., 2009), cartilaginous fish (Hyodo et al., 2004; Gwee et al., 2009), teleosts (Heierhorst et al., 1989; Morley et al., 1990; Hyodo et al., 1991; Suzuki et al., 1992; Venkatesh and Brenner, 1995; Warne et al., 2000), lungfishes (Hyodo et al., 1997), coelacanth (Gwee et al., 2008), amphibians (Nojiri et al., 1987; Searcy et al., 2009), reptiles (Montefiano et al., 2001), and birds (Hamann et al., 1992). This has complemented earlier works of characterization of peptide principles of the neurohypophysis by chromatographic methods and has provided useful insights into the distribution of the peptides in different vertebrate species, their precursor structure and evolution. The nonapeptides are synthesized as part of a large precursor molecule that undergo post-translational processing to release the nine amino acid hormone with two half cysteine residues at the first and sixth positions forming a disulphide bridge, and a cysteine- rich protein called neurophysin (Acher, 1996). Neurophysin aids in proper folding of the precursor and its sorting into secretory vesicles (de Bree et al., 2000). In vertebrates, about 15 nonapeptides are known till date. They are classified into two families on the basis of the amino acid at the 8th position, as the basic and neutral peptide families. Each vertebrate possesses at least two peptides, one each from the two families except cyclostomes, where only the basic peptide representative vasotocin (VT) is documented (Gwee et al., 2009). This has led to a widely accepted theory that the VT precursor gene is the ancestor of all vertebrate nonapeptide precursor genes, and has given rise to the two lineages following an event of duplication early in the vertebrate evolution probably at the base of the gnathostome lineage. Evolutionarily too, the two peptide families follow distinct lineages with VT representing the common basic peptide in all nonmammalian vertebrates to be replaced by vasopressin (VP) in mammals, while isotocin (IT) is the neutral peptide in teleosts, mesotocin (MT) in lobe-finned fishes and noneutherian tetrapods, and oxytocin (OT) in eutherians. In elasmobranchs, a host of different neutral peptide homologs has been reported which is in contrast with the remarkable stability in the type and numbers of nonapeptides in any other group of Vertebrata (Acher, 1996). Presence of more than two neurohypophyseal peptides has been reported from groups like elasmobranchs, amphibians, and metatherians (Chauvet et al., 1983, 1984, 1985; Rouille et al., 1988, 1989; Parry et al., 2000; Hyodo et al., 2004). In teleosts, VT and IT have been reported as the basic and neutral hormone representatives, respectively. Larhammar et al. (2009) emphasized the need for simplifying the nomenclature with the original names VP for the basic peptides and OT for all neutral peptides.

The two evolutionary lines of basic and neutral nonapeptides across vertebrates represent a contrasting picture in the frequency of substitutions in the 9 amino acid (aa) hormone. In the basic lineage, there have been lesser substitutions than that in the neutral line, as evident from the distribution of the peptides in vertebrates. In fact, the number of cognate receptors for the two peptide families is also contrasting. While the stable basic nonapeptide line has a diverse repertoire of at least five types of cognate receptors (V1A, V1B, V2A, V2B, and V2C), the neutral nonapeptide series has a single type (OTR) in all vertebrate groups (Ocampo Daza et al., 2012; Yagamuchi et al., 2012; Lagman et al., 2013). All types of receptors are present in the early gnathostomes, the cartilaginous fish (Yagamuchi et al., 2012) suggesting that the ligand-receptor selectivity might have been established very early in the vertebrate evolution and it is the co-evolution of the hormone with its cognate receptors which has maintained the two families of basic and neutral nonapeptides, and allowed only a basic amino acid substitution at the 8th position in the basic line and a neutral amino acid substitution at the 8th position in the neutral line. While, the ligand- receptor selectivity might have directed the evolution only in the 9 aa hormone part; the larger neurophysin region is not under this evolutionary pressure. Therefore, to know the evolutionary history of the NH precursors especially in the light of recurrent local and global duplication events occurring throughout the vertebrate history, there is a need to do a phylogenetic study over the entire precursor rather than considering the distribution of peptides in different vertebrate groups.

Teleosts had undergone a third round of whole genome duplication, (3R) (Meyer and Schartl, 1999; Christoffels et al., 2004; Jaillon et al., 2004) in addition to the two vertebrate-specific whole genome duplications. After the 3R in teleosts, there was diploidization by the loss of 3R paralogs. These losses of the 3R paralogs were different in various groups of teleosts (Garcia de la serrana et al., 2014). Even in such a scenario, the consistency in the number and type of nonapeptides in teleosts is noteworthy. However, the teleost NH precursors available in the public database are mostly sequences from the species-rich superorder Acanthopterygii while only few nonapeptide precursor sequences from the other species-rich group, the superorder Ostariophysi are available. The Acanthopterygii and Ostariophysi have undergone independent adaptive radiations (Santini et al., 2009), and are known to have suffered differential losses of 3R paralogs. Therefore, it is pertinent to have sequence information from more species representing all groups in Teleostei, particularly the superorder Ostariophysi to understand the evolution of the NH precursor genes in teleosts.

Previous studies from our laboratory have shown a well-developed and highly organized VT system in the Asian air-breathing catfish *Heteropneustes fossilis*. VT was characterized in both brain and ovary of the catfish with seasonal changes (Singh and Joy, 2008), and has been shown to be involved in reproductive functions such as steroidogenesis, oocyte maturation, oocyte hydration, ovulation and prostaglandin secretion (Singh and Joy, 2009b, 2010, 2011; Joy and Singh, 2013). IT is less effective in influencing these events. Three VT receptors have been cloned from the catfish V1a1 (accession no.KF434615.1), V1a2 (accession no.KF434616.1), and V2A (accession no.KF434617.1), which display a wide tissue distribution (Rawat et al., 2015). All the three receptors are expressed in the brain and ovary, which are the two major sites of VT production.

The catfish *H. fossilis* holds an important phylogenetic position as it is believed to be the sole survivor of an old clade that went extinct at the K-T (Cretaceous-Tertiary transition period) boundary (Jansen et al., 2006) when a major extinction event took place due to severe volcanic activity (Deccan traps) in the Asian region. This might have severely polluted and deoxygenated the inland water bodies and only fish with air-breathing apparatus survived. Air-breathing habit also demands a shift in osmoregulation and fluid homeostasis in which apart from other systems, the NH nonapeptides play a key role. In view of the important taxonomic and phylogenetic position of *H. fossilis* and its air-breathing habit, molecular characterization of the NH peptide genes as a follow on of earlier anatomical and physiological studies on VT has been attempted in the present work. The sequence information was also extended to *Clarias batrachus*, another air- breathing catfish, and used in the phylogenetic analysis. The catfish NH precursor sequence information from the present study will add to the information pool of teleost nonapeptides from a group other than the superorder Acanthopterygii and allow discussing the phylogeny of the vertebrate NH nonapeptides in the light of 3R and subsequent diploidization in teleosts. A tissue distribution profile for these genes was also carried out since VT was described in the catfish ovary earlier.

## Materials and methods

### Animal collection and acclimation

Adult *H. fossilis* (40–50 g) were collected from local fish markets in Varanasi. They were maintained in the laboratory for 48 h under natural photoperiod (13.0 L: 11.0 D) and temperature (25 ± 2°C) to overcome stress due to transportation and fed daily with goat liver *ad libitum*. Adult *C. batrachus* were obtained in the resting phase for the cloning work. The fish were weighed and sacrificed by decapitation. All experiments were performed in accordance with the guidelines of the Animal Ethics Committee, Banaras Hindu University, Varanasi.

### Chemicals and reagents

Guanidine thiocyanate—phenol solution (Qiagen), Revert-Aid H Minus First Strand cDNA Synthesis Kit (Fermentas), 2X PCR Master Mix (Fermentas), DNase (Ambion) and veriquest SYBR green qPCR master mix (affymetrix) were purchased through local suppliers. Agarose, Tris base, Glacial acetic acid, EDTA–Na2 and other chemicals were of molecular grade, purchased from E-Merck, India. LB broth, LB Agar, ampicillin, X-Gal and IPTG were purchased from Himedia, India. Hyaluronidase type IV was purchased from Sigma-Aldrich, India. The primers used were synthesized by Integrated DNA Technology, India.

### Cloning of VT and IT genes from *H. fossilis* and *C. batrachus*

For cloning of VT, an approach of first isolating partial VT cDNA using degenerate PCR (polymerase chain reaction) followed by 3′ and 5′ Rapid Amplification of cDNA ends (RACE) to obtain the full length VT cDNA sequence was applied. Total RNA from the brain of acclimatized female catfish in the resting phase of the reproductive cycle was prepared by the single-step method of RNA isolation (Chomczynski and Sacchi, 1987) by acid guanidium thiocyanate-phenol-chloroform extraction using Qiazol (Qiagen) as the monophasic lysis buffer. Five μg total RNA was reverse- transcribed using random hexamer primers and Revert Aid M-MuLV reverse transcriptase in a 20 μL reaction volume (first strand cDNA synthesis kit, Fermantas) using the manufacturer's protocol. One μL of the resulting cDNA was used to amplify partial VT cDNA in 25 μL reaction volume using 2 X PCR master mix (Fermentas) and VT degenerate forward primer and VT degenerate reverse primer in a cycling condition of one cycle of 95°C, 5 min; then 35 cycles of 95°C, 30 s; 57°C, 30 s; 72°C, 1 min; and a final 7-min elongation at 72°C. The degenerate primers were designed using the software iCODEHOP (COnsensus-DEgenerate Hybrid Oligonucleotide Primers), in which teleost VT precursor sequences were used as inputs (Table 1). The resulting 145 bp partial VT amplicon was purified using Nucleo-pore PCR clean- up gel extraction kit (Genetix) and sequenced taking services of Eurofin genomics, Bangalore, India. For obtaining full length sequence, 3′ RACE was done for which total RNA from the brain was reverse-transcribed using oligo dT anchor primer and Revert Aid M-MuLV reverse transcriptase. One μL of the resulting cDNA was amplified using a VT H FP (corresponding to the hormone moiety), designed from the partial sequence and anchor primer to obtain a 700 bp 3′ RACE amplicon, which was cloned and sequenced. This sequence was utilized for designing two gene-specific nested reverse primers to do 5′ RACE. Total RNA from the brain was reverse-transcribed using the outer gene specific VT RP and Revert Aid M-MuLV reverse transcriptase. The resulting cDNA was purified using the Nucleo-pore PCR clean- up gel extraction kit (Genetix) to remove residual dNTPs. The purified cDNA was dA tailed using dATP and terminal transferase. The dA tailed cDNA was used for PCR amplification using the nested RP 2 primer and oligo dT anchor primer. The 5′ RACE amplicon was purified, cloned and sequenced. Full length VT cDNA sequence was submitted to the GenBank with the accession no. JX035928.1

**Table 1:** **List of primers used for cloning and qPCR of vasotocin and isotocin precursors**.

**Primers**	**Sequences**
VT d FP	TCCGCTTGTTACATCCARAAYTGYCC
VT d RP	ACATCCCAGTCCCTCTCCRCARCDAT
VT H FP	GTTACATCCAGAACTGCCCCAGA
VT FP	TGTTACATCCAGAACTGCCCCAGA
VT RP	CAGCCCAGTCCTTCTCCACAGCA
RP1	TTTATCTCCAGGACCGCAAG
RP2	CAGGACCGCAAGACATACAC
VT UTR FP	GTCCAGTGAGAGACAGACCTCCGG
IT H FP	ACATCTCCAACTGTCCCATC
IT UTR FP	CATCAGCTACTGAAGCTACTGATTCGT
Oligo dT anchor primer	GACCACGCGTATCGATGTCGACTTTTTTTTTTTTTTTTV
Anchor primer	GACCACGCGTATCGATGTCGAC
IT FP	TCAATCTTCTGCATGCTGTGTCT
IT RP	CACACGCCATGCACTGTCTATTG
β actin FP	TGGCCGTGACCTGACTGAC
β actin RP	CCTGCTCAAAGTCAAGAGCGAC

For cloning IT precursor cDNA, total RNA from the brain was reverse-transcribed using an oligo dT anchor primer and Revert Aid M-MuLV reverse transcriptase. One μL of the resulting cDNA was PCR amplified using a sense or forward primer IT H FP designed from the IT hormone moiety and anchor primer, following the 3′ RACE protocol. The resulting 700 bp amplicon was sequenced taking services from Eurofin genomics. To get the 5′ end of the precursor cDNA, 5′ RACE was done. Total RNA from the brain was reverse- transcribed using an outer reverse or antisense primer, RP1 (designed over a conserved region of the neurohypophyseal peptide neurophysin) and Revert Aid M-MuLV reverse transcriptase. The resulting cDNA was purified and dA tailed. The dA tailed cDNA was used for PCR amplification using an overlapping and nested antisense primer RP2 and oligodT anchor primer. The resulting 200 bp 5′ RACE amplicon was ligated into pGEMT vector (promega kit), following the manufacturer's protocol and transformed into the *E. coli* DH5 α competent cells. Transformed cells were plated into LB Agar solid medium containing ampicillin, X- Gal and IPTG to select positive transformants and blue- white screening was done for recombinant colonies. Plasmids from white colonies (colonies transformed with recombinant plasmids) were extracted by alkaline lysis method and sequenced. Sequence information from the IT precursor containing inserts (as known by *in silico* translation of the insert sequences) was used to design a sense primer from the 5′ UTR region (IT UTR FP), followed by 3′ RACE with this primer to obtain the entire IT precursor sequence, which was submitted to the GenBank with the accession no. JX669009.1.

Partial sequences of *C. batrachus* VT and IT precursors were obtained by 3′ RACE with the forward primers VT H FP and IT H FP (the same used for *H. fossilis*), to obtain a sequence read from the hormone region till the 3′ untranslated region. The primer details are given (Table 1).

### Sequencing of VT and IT transcripts from ovary

Since the VT and IT transcripts are less abundant in the ovarian follicles, a semi-nested 3′ RACE was done to get a single, sharp amplification of the gene transcripts. Briefly, it included a primary PCR using VT UTR FP, IT UTR FP spanning the 5′ UTR region of VT and IT genes, respectively and anchor primer to amplify 1 μL of follicular cDNA (made by reverse transcription of RNA using oligodT anchor primer), followed by a secondary PCR using VT H FP, IT H FP and anchor primer to amplify1 μL of the primary PCR product. The amplicons from the secondary PCR was purified and sequenced.

### Data mining for phylogenetic analysis

The NH nonapeptide precursor sequences of species representing most vertebrate classes were tried to be included in the phylogenetic analysis. The sequences were procured from the GenBank. Details of the sequences of the species with accession numbers are given in Supplementary Table 1. For procuring the NH precursors of *Ictalurus punctatus*, a BLAST search against the *Ictalurus* EST database (Lu et al., 2010) was done using the *H. fossilis* VT and IT sequences as queries. The identities of the putative VT and IT precursor mRNAs obtained were confirmed by *in silico* translation of the sequences. The spotted gar *Lepisosteus oculatus*, Holostei is a basal actinopterygian species representing a pre 3R genome. The neurohypophyseal nonapeptide precursor sequences of this species were procured by BLAST searches in its Nucleotide database and analyzed.

### Phylogenetic analysis

Phylogenetic trees were constructed using the Neighbor- Joining (NJ) and Maximum-Likelihood (ML) method. Evolutionary distances were computed using the Poisson correction method for rate of amino acid substitution. All phylogenetic studies were done using the software MEGA 6 (Tamura et al., 2013). In addition to the calculation of evolutionary distance of precursor sequences within and between species, the sequences were grouped according to the taxonomic units for calculation of evolutionary distance between groups (Table 2). Keeping in view of the enormous species diversity, the teleost sequences of the two superorders Acanthopterygii and Ostariophysi were independently treated. Multiple sequence alignment used for the tree construction and distance calculation was the same and was done in MEGA6 using Clustal W parameters. Gonnet protein weight matrix with gap opening and gap extension penalties of 10 and 0.2 respectively was used for the alignment. The alignment is provided in a Supplementary data sheet 1.

**Table 2:** **Details of the taxonomic groups and the species included in each for the phylogenetic distance calculation between groups**.

**S. No**	**Taxonomic group**	**Species**
1	Cyclostome VT	*Lethenteron camtschaticum*, *Eptatretus burgeri*
2	Acanthopterygii VT	*Thalassoma bifasciatum*, *Parajulis poecilepterus*, *Halichoeres trimaculatus*, *Sparus aurata*, *Takifugu rubripes*, *Platichthys flesus*, *Epinephelus coioides*, *Amphiprion melanopus*, *Oryzias latipes*, *Cyprinodon variegates, Haplochromis burtoni*
3	Ostariophysi VT	*Heteopneustes fossilis*, *Clarias batrachus* and *Ictalurus punctatus, Danio rerio, Astyanas mexicanus* (LOC103042813, LOC103030472)
5	Cartilaginous fish VT	*Triakis scyllium*, *Callorhinchus milli*
6	Lungfish VT	*Neoceratodus forsteri*, *Protopterus annactens*
7	Coelacanth VT	*Latimeria menadoensis*
8	Acanthopterygii IT	*Platichthys flesus*, *Sparus aurata*, *Halichoeres trimaculatus*, *Parajulis poecilepterus*, *Takifugu rubripes, Oryzias latipes, Xiphophorus maculatus, Amphiprion melanopus, Haplochromis burtoni*
8	Ostariophysi IT	*Heteopneustes fossilis*, *Clarias batrachus*, *Ictalurus punctatus, Danio rerio, Asatyanax mexicanus* (LOC103043649, LOC103044969)
10	Cartilaginous fish neutral hormone precursors	*Triakis scyllium phasitocin*, *Triakis scyllium asvatocin*, *Torpedo marmorata isotocin*, *Callorhinchus milii oxytocin*
11	Lungfish MT	*Neoceratodus forsteri*, *Protopterus annectens*
12	Coelacanth MT	*Latimeria menadoensis*
12	Amphibian VT	*Bufo japonicus*, *Plethodon shermani*, *Taricha granulosa*, *Typhlonectes natans*
13	Amphibian MT	*Bufo japonicus*, *Typhlonectes natans*, *Taricha granulosa*
14	Reptilian VT	*Podarcis siculus*
15	Reptile MT	*Podarcis siculus*
16	Avian VT	*Taeniopygia guttata*, *Coturnix coturnix*, *Gallus gallus*
17	Avian MT	*Taeniopygia guttata*
18	Mammalian VP	*Homo sapiens*, *Rattus norvegicus*
19	Mammalian OT	*Homo sapiens*, *Rattus norvegicus*

*The accession numbers of the sequences are given in Supplementary Table 1*.

### Analysis of VT and IT genomic location and synteny analysis

The nonapeptide gene-containing chromosome blocks of spotted gar and representative teleost species were obtained from the NCBI and Ensembl genome browser. Fugu, medaka, tilapia, and stickleback belong to the teleost superorder Acanthopterygii, and zebrafish and cavefish belong to the superorder Ostariophysi. The selection of the chromosome blocks from Ensembl database was based both on searching the genome assemblies for the nonapeptide genes by named searches as well as BLAST searches using teleost nonapeptides as queries. This is to ensure that nonapeptide loci are obtained even in the species where the genes are not annotated. The chromosome blocks were obtained from the NCBI database mostly by navigating the genomic context of the genes. Blocks obtained from both Ensembl and NCBI were tallied for each species and a consensus assembly of genes on the blocks was arrived at by filling in for the nonannotated genes in one of the databases from information obtained from the other. Apart from the nonapeptide genes, the assemblies were also searched for the genes linked with the nonapeptide genes and the additional chromosomal blocks harboring these genes (paralogous to the linked genes), if distinct from the nonapeptide loci. Additionally, the loci of the linked genes in human were obtained and used as a reference tetrapod assembly for comparison of conserved synteny.

### Analysis of VT and IT precursor gene expression

A two step qPCR was conducted to show tissue and seasonal expression of VT and IT genes. For tissue expression profile, various tissues viz. brain, gills, liver, muscle, kidney, and gonads were collected. For the ovarian tissue sample, the follicles were collected from the post-vitellogenic ovary and the follicular layer was separated from oocyte using hyaluronidase treatment (Mishra and Joy, 2006). Briefly, the protocol included treatment of a batch of about 500 follicles with 0.3% hyaluronidase (Type IV) for 5 min. The isolated follicular envelope (granulosa and theca cells) and the denuded oocytes were used for the expression studies. For seasonal expression, brain and ovary was collected during the different reproductive phases, resting (November–January), preparatory (February–April), pre-spawning (May–June), spawning (July–August) and post-spawning (September–October). About 100 mg tissues were used for extraction of total RNA by the single-step method of RNA isolation. To remove genomic DNA contamination from the preparation, DNAase (Ambion) treatment (2 units/10 μg RNA) was given and subsequently DNAase was heat inactivated at 75°C in presence of EDTA. RNA purity was checked by A_260_/A_280_ ratio. Samples having a ratio above 1.8 were only considered for reverse transcription. Two μg of the total RNA was reverse-transcribed using random hexamer primers and Revert Aid M-MuLV reverse transcriptase in a 20 μL reaction volume (first strand cDNA synthesis kit, Fermantas) using the manufacturer's protocol. The resulting cDNA was diluted 10 times and 1 μL was used in a PCR reaction of 20 μL containing veriquest SYBR green 2X master mix and VT/IT FP and IT FP/ RP using manufacturer's protocol in an Applied Biosystem 7500 machine with a thermal condition of 50°C for 2 min, 95°C for 10 min, followed by 40 cycles of 95°C for 15 s and 60°C for 1 min. The specificity of the PCR product was checked by dissociation curve analysis of the amplicon and was also checked by agarose gel electrophoresis. The relative gene expression in different tissues and across different reproductive seasons was expressed using the comparative CT method with the catfish β- actin (accession number FJ409641.2), used as the endogenous control. The resting phase brain cDNA was taken as the calibrator sample for relative quantity calculation (Livak and Schmittgen, 2001). Each reaction was set up in duplicate and the average CT value was taken for calculation. Graphs were plotted with the mean RQ (relative quantity) values (2^−ΔΔ*CT*^) of five fish and represented as mean ± SEM. The results were analyzed by One-Way ANOVA (*p* < 0.001), followed by Newman-Keuls' test (*p* < 0.05) for statistical significance.

## Results

### Sequence analysis of VT and IT precursors from *H. fossilis*

**Figure 1:**
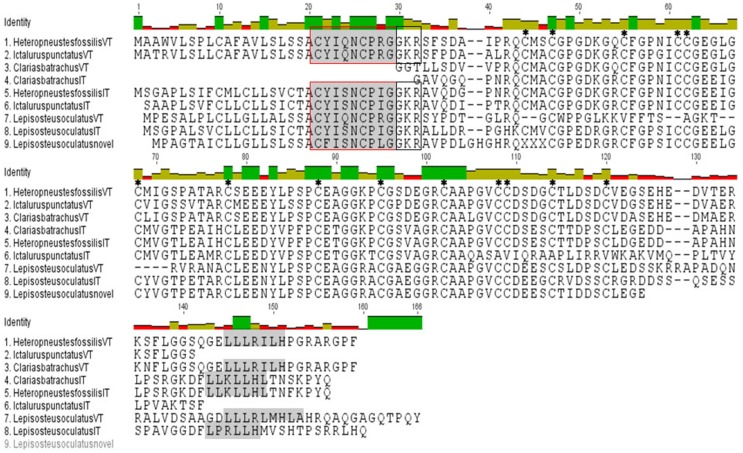
**Multiple sequence alignment of predicted structures of VT and IT precursors of *H. fossilis, C. batrachus, I. punctatus*, and spotted gar**. Hormone moiety and leucine- rich box in the C-terminal neurophysin are shaded and “GKR” (enzymatic processing signal) is boxed. The cysteine residues have been star marked. The *C. batrachus* sequences are partial at the N-terminal without the signal peptide and hormone moiety.

The isolated full length cDNA of the HfVT precursor is 618 bp long with a cds from 60 to 524 bp and encodes a VT precursor of 155 amino acids (aa; Figure 1; Supplementary Figure 1A). The deduced VT precursor codes for a signal peptide (1–20 aa), a 9 aa hormone moiety (21–29 aa), a 3 aa enzymatic processing site (30–32 aa) and a 122 aa neurophysin (34–155 aa). The central part of the neurophysin (40–118 aa) is highly conserved and belongs to the hormone_5 superfamily (NCBI CDD accession *pfam00184*) of the neurohypophyseal hormones (NCBI CDD accession *smart00003*). There are 14 cysteine residues in this region (42, 45, 53, 59, 60, 66, 76, 86, 93, 100, 106, 107, 112, and 118). The C- terminal part of the neurophysin (119–155 aa) is poorly conserved except for the presence of a leucine-rich region “LLLRILH” (141–146 aa) (Figure 1) that is conserved across all vertebrate groups. All these features conform to the typical features of the neurohypophyseal hormones. There is no putative site for N- linked glycosylation in C-terminal domain corresponding to copeptin, making it akin to other teleost neurohypophyseal hormone precursors, unlike lungfish and tetrapod hormone precursors.

The isolated HfIT precursor cDNA is of 708 bp long with a cds from 56 to 508 bp and encodes the hormone precursor of 151 aa (Figure 1; Supplementary Figure 1B). Similar to the VT precursor, the putative IT precursor encodes for a signal peptide (1–20 aa), a hormone moiety of 9 aa (21–29 aa), enzymatic processing signal (30–32 aa) and a 118 aa neurophysin (34–151 aa). Further, the central part of neurophysin (40–118 bp) is highly conserved and belongs to the hormone_5 superfamily (NCBI CDD accession pfam00184) of the neurohypophyseal hormones (NCBI CDD accession smart 00003) and there are 14 cystine residues in this region (42, 45, 53, 59, 60, 66, 76, 86, 93, 100, 106, 107, 112, 118) (Figure 1). The presence of an extended C-terminal part homologous to the C- terminal part of the VT precursors and the mammalian vasopressin-associated copeptin makes it similar to other IT precursor but dissimilar to other neutral peptide precursors like MT and OT precursors and other cartilaginous fish neutral hormone precursors which lack an extended C-terminal region. The C- terminal part of the neurophysin (119–151 aa) is poorly conserved except for the presence of a leucine- rich region “LLKLLHL” (138–144 aa), which is a signature of the C-terminal region of the neurohypophyseal hormone precursors. There is neither a dibasic cleavage site nor putative site for N- linked glycosylation in this region similar to the VT precursor.

### Sequence analysis of other catfish VT and IT precursors

The cloned partial sequences of *C. batrachus* (Cb) VT and IT cDNA extend from the N-terminal neurophysin coding region to the 3′ UTR (Supplementary Figures 1C,D). The putative precursors, like the *H. fossilis* precursors, have all the essential features of the neurohypophyseal precursor proteins, with a central conserved neurophysin having 14 cysteine residues. The poorly conserved C- terminal regions have leucine-rich boxes, “LLLRILH” in the VT precursor and “LLKLLHL” in the IT precursor (Figure 1).

*In silico* translation of the clones, recognized as the neurohypophyseal hormone precursors from the EST database of *I. punctatus* (Ip) by BLAST reveals that they indeed code for VT and IT precursors. The sequence read of the putative VT precursor clone (acc. no. BM495247) starts from 5′ UTR region and ends in the C-terminal part of the neurophysin. On the other hand, the putative IT precursor clone (acc. no. BE213165) sequence extends from the signal peptide region to the neurophysin (Supplementary Figures 1E,F and Figure 1).

The 3′ UTR region of the *H. fossilis* VT gene has a long stretch of 19 CA repeats. The *C. batrachus* VT gene has only 4 CA repeats in the 3′ UTR at approximately the same position as the *H. fossilis* VT gene (Supplementary Figures 1A,C).

### Analysis of spotted gar neurohypophyseal nonapeptides

BLAST search revealed that the spotted gar has three neurohypophyseal nonapeptides, VT as the basic nonapeptide (XM 006626529.2) and two nonapeptides in the neutral family, IT (XM 006626499.1) and a novel peptide (XM 006626523.1, 1–366 nt) with a unique hormone “CFISNCPLG” is present. While the spotted gar IT has an extended C-terminal with a leucine- rich core, the novel nonapeptide has a short C-terminal like all other neutral nonapeptide precursors of vertebrates (Figure 1).

### Tissue and seasonal expression of VT and IT precursor genes

**Figure 2:**
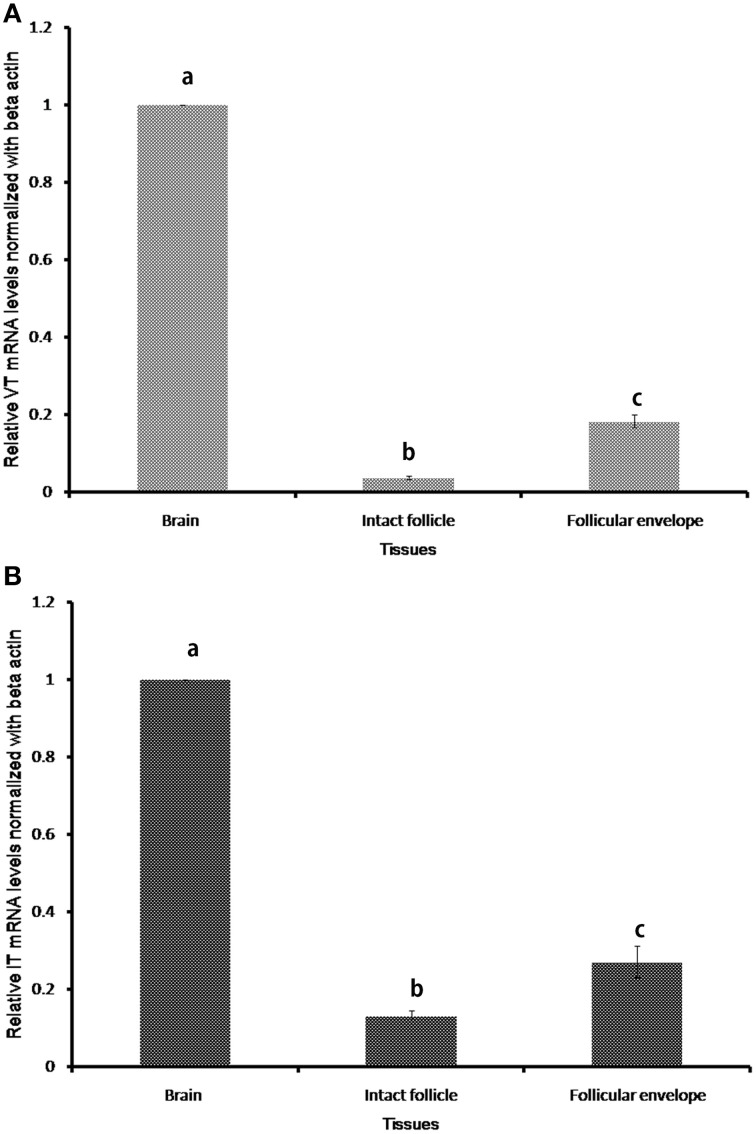
**Relative tissue expression levels of VT precursor (A) and IT precursor (B)**. Graphs were plotted with the mean RQ values (2^-ΔΔ*CT*^) of expression in brain, intact follicle and follicular envelope. The RQ values (mean ± SEM; *n* = 5 fish each) were calculated with the resting phase brain cDNA as the calibrator. Groups with different alphabets show significant variations in the expression levels.

The nonapeptide genes expressed only in the brain and ovary of the catfish (Figure 2). In the ovary, the expression was confined to the follicular envelope and not in oocytes. There was no expression in the gill, liver, kidney, testis and muscle. The VT and IT mRNA expressions were significantly higher in the brain than the ovary (Figure 2).

The sequence reads of the semi-nested 3′ RACE amplicons of VT and IT in the ovary were partial but good enough to indicate the presence of functional transcripts of the genes in ovarian follicles (not shown). Also, the sequences were similar to the brain transcripts, pointing to the same gene responsible for synthesis of the nonapeptides in both brain and ovary.

**Figure 3:**
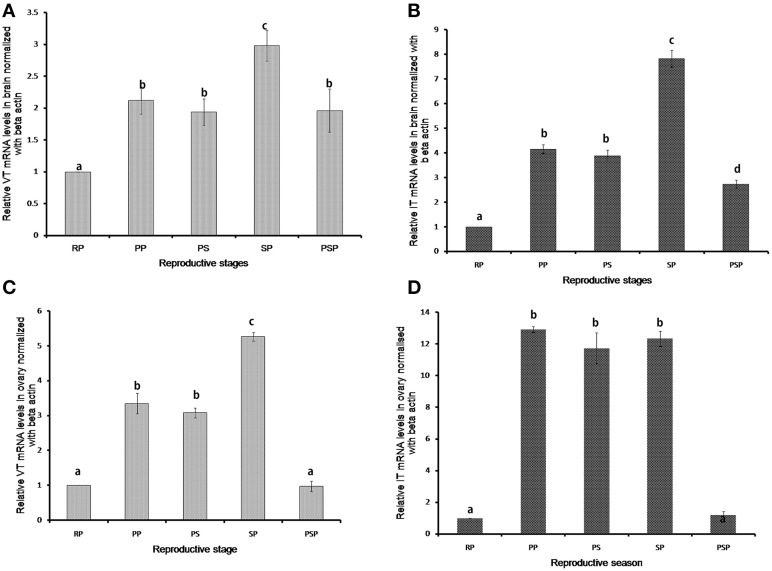
**Seasonal expression levels of VT and IT precursors in brain (A,B) and ovary (C,D)**. The RQ values (2^-ΔΔ*CT*^) were calculated with the resting phase brain or ovary as the calibrator samples (mean ± SEM; 5 fish each). Groups with different alphabets show significant variations in the expression levels. RP, resting phase; PP, prepratory phase; PS, pre-spawning phase; SP, spawning phase; PSP, post-spawning phase.

VT and IT expressions showed significant seasonal changes both in the brain and ovary (Figure 3). In the brain, the VT expression was low in the resting phase and increased in the preparatory, pre-spawning and spawning phases. The expression remained high in the post-spawning phase similar to the preparatory and pre-spawning phases. The expression of IT precursor was similar to that of the VT precursor but it decreased sharply in the post-spawning phase as in the resting phase.

The highest expression was observed in the spawning phase for both the genes (Figures 3A,B). In ovary too, the expression of both VT and IT increased in the reproductive phases compared to the resting phase (Figures 3C,D). However, while in the spawning phase the expression of the VT precursor was higher as compared to the preparatory and pre-spawning phases (Figure 3C), the IT precursor expression remained equally high in all the reproductive phases (Figure 3D).

### Evolutionary history of teleost NH peptide precursor genes

#### Phylogenetic analysis

**Figure 4:**
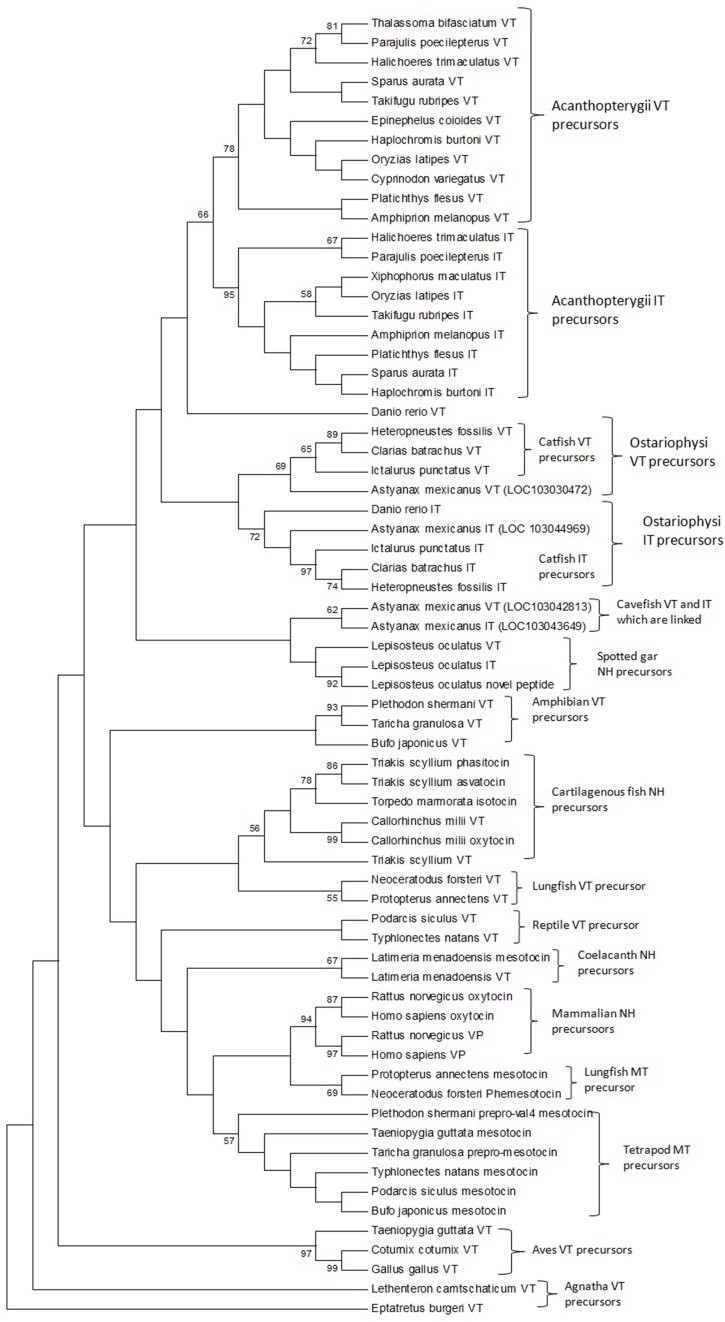
**The phylogenetic tree of vertebrate neurohypophyseal nonapeptides constructed by the Maximum-Likelihood method**. The bootstrap consensus tree inferred from 500 replicates is taken to represent the evolutionary history of the taxa analyzed. The analysis involved 67 amino acid sequences. All positions with less than 95% site coverage were eliminated. There were a total of 97 positions in the final dataset. Evolutionary analyses were conducted in MEGA6. References to the sequence and species name are given in Supplementary Table 1.

**Figure 5:**
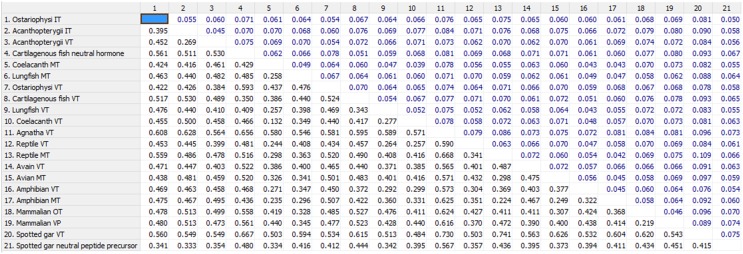
**Shows the number of amino acid substitutions per site from averaging overall sequence pairs between groups**. Standard error estimate(s) shown above the diagonal were obtained by a bootstrap procedure (500 replicates). Analyses were conducted using the Poisson correction model (Zuckerkandl and Pauling, 1965). The analysis involved 67 amino acid sequences. All positions with less than 95% site coverage were eliminated. That is, fewer than 5% alignment gaps, missing data, and ambiguous bases were allowed at any position. There were a total of 97 positions in the final dataset. Evolutionary analyses were conducted in MEGA6 (Tamura et al., 2013).

The phylogenetic inference on vertebrate NH peptide precursors was drawn from both tree constructions as well as from the evolutionary distance calculation in between groups. Both the NJ and ML trees are similar and only the ML tree is shown in Figure 4. The NH sequences of salmonids and catastomids (teleosts) were included in the initial analysis but were removed from the tree as they did not make a definite cluster probably due to polyploidization that led to two copies of VT and IT genes, and gene conversions (Hyodo et al., 1991; Suzuki et al., 1992). Both the phylogenetic tree and evolutionary distance calculation in between the groups (Figure 5) show that the NH precursor paralogs of cartilaginous fish, coelacanth and mammals are phylogenetically closer than their homologs in other groups. The cartilaginous fish VT and neutral hormone precursors share a distance of 0.350, coelacanth VT and MT precursor share a distance of 0.132, and the mammalian VP and OT precursors share a distance of 0.219. The evolutionary distances of the NH precursors of these groups with that of other vertebrate groups are larger (Figure 5). Amongst the cartilaginous fish, elephant shark VT and OT precursors share a closer phylogenetic relation, as shown by the ML tree.

The phylogenetic tree with the VT precursor of Agnatha as the outgroup also shows that the teleost VT and IT precursors form a cluster with those of the spotted gar (Holostei) while other gnathostome NH precursors form another cluster, which includes the cartilaginous fish VT and neutral hormone precursors, and coelacanth and lungfish VT and MT precursors. However, the bootstrap support for the node separating the teleost NH precursors and the other gnathostome NH precursors is low.

The catfish NH precursors expectedly segregated into their respective clusters, i.e., the HfVT, CbVT, IpVT and HfIT, CbIT, IpIT, making two distinct clusters. Within these clusters, the CbVT, HfVT and CbIT, HfIT are closer to each other than to IpVT and IpIT, respectively. The spotted gar NH precursors made a common cluster; the two neutral NH peptide precursors (IT precursor and the novel peptide precursor) are evolutionarily closer than the VT precursor. The Acanthoperygii VT and IT precursors are phylogenetically closer than their orthologs in the Ostariophysi. The evolutionary distance in between the groups confirms this. The evolutionary distance between the Acanthopterygii VT and IT precursors is only 0.269 while that between Acanthopterygii VT and Ostariophysi VT precursors is 0.426, and the Acanthopterygii IT and Ostariophysi IT precursors is 0.452. The ostariophysian VT precursor form a cluster having the catfish VT precursors and one of the two VT precursors of cavefish (*Astyanax mexicanus*), while zebrafish (*Danio rerio*) VT precursor does not form part of this cluster. The ostariophysian IT precursors make a cluster that includes the catfish and zebrafish IT precursors, and one of the IT precursors of the cavefish. The other cavefish VT and IT precursors, which do not form a part of the ostariophysian clusters, make a separate cluster and share a close phylogenetic relation with each other. Unlike the Acanthopterygii VT and IT precursors, the Ostariophysi VT and IT precursors are phylogenetically distant and share an evolutionary distance of 0.422 (Figure 5).

#### Linkage and synteny analysis of VT and IT in teleosts and spotted gar

**Figure 6:**
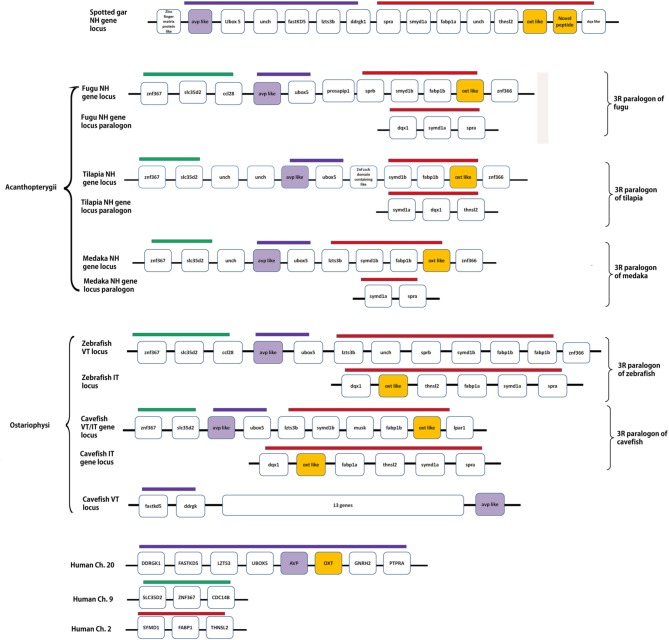
**A schematic representation of the genomic loci of spotted gar and teleost NH precursor genes and their paralogons**. Human assembly is represented as a reference of tetrapod genomic context of the respective loci. The figure shows the linked situation of the genes in spotted gar (Holostei), acanthopterygian teleosts and one of the loci in cavefish, and unlinked situation in teleosts belonging to Ostariophysi. Conserved syntenic blocks have bars with the same colors. Details of genes and proteins encoded are: *ubox5*, ring finger protein 37; *fast KD5*, fast kinase domain containing protein5; *lzts3b*, leucine zipper, putative tumor suppressor family 3b; *DDRGK1*, DDRGK domain containing protein1; *spra*, sepiapterin reductase; *smyd1*, SET and MYND domain containing protein; *fabp1*, fatty acid binding protein1; *thnsl2*, threonine synthase like; *avp*, arginine vasopressin; *oxt*, oxytocin; *znf367*, zinc finger domain protein 367; *slc35d2*, UDP- N- acetylglucosamine transporter; *ccl28*, c-c motif chemokine 28; *prosapip1*, prosap interacting protein 1; *dqx*, DEAQ box RNA dependent ATPase; *znf366*, zinc finger protein 366; *ptpra*, protein phosphatase receptor type A, *GnRH2*, gonadotropin releasing hormone 2; *cdc14b*, cell divison cycle 14b; unch, uncharacterized gene.

Details of the chromosome blocks and linkage groups used in the analysis are summarized in Supplementary Table 2. A schematic representation of conserved synteny in the spotted gar and teleosts is presented in Figure 6. In the spotted gar, VT, IT and the novel peptide precursors are present in the same linkage group. But, the VT precursor gene (*avp*-like) and IT precursor genes (*oxt*-like) are not arranged in tandem, unlike in human and *Xenopus* where the nonapeptide genes are present in tandem. However, the *oxt*-like gene and the novel peptide precursor gene (both in the neutral family) are arranged in tandem. Similarly, in acanthopterygian teleosts (fugu, tilapia, medaka, sticleback), the *avp*- like (VP- like) and *oxt*- like genes are in the same linkage group but are not present in tandem. In the ostariophysian teleosts, two contrasting situations exist. In zebrafish, *avp*- like and *oxt*- like are present on two different chromosomes. In contrast, in the cavefish where there are two genes each for VT and IT, one pair is present in the same linkage group like in the acanthopterygian fish. The other *avp*-like and *oxt*-like genes are present on different linkage groups so that in the cavefish, there are three linkage groups harboring the nonapeptide genes. The chromosome block in which the two genes are linked shares a conserved synteny with the acathopterygian NH loci and zebrafish *avp*-like containing linkage group. The other *oxt*-like gene is present in the paralogon of this block and shares a conserved synteny with the zebrafish *oxt*-like containing block and the Acanthopterygii paralogon of the NH loci. The genomic context of the other *avp*-like gene is considerably different from that of the teleost and spotted gar nonapeptide-harboring chromosome blocks or their paralogons. But, on a closer examination it seems to share a conserved synteny with the tetrapod nonapeptide loci as it is present in the same linkage group as the genes *fast kd 5* and *ddrgk* (genes linked with NH genes in tetrapods) but separated from them by 13 genes.

## Discussion

### Catfish VT and IT precursor genes and proteins

In the present study, we cloned a full length cDNA of 618 bp from *H. fossilis* that coded for a VT precursor of 155 aa. There is no difference in the sequence of the brain and ovarian transcripts and hence the same gene expresses in both the tissues. We could also clone *C. batrachus* partial VT precursor gene from the N-terminal neurophysin coding region to the 3′ UTR. The catfish VT precursor gene does not show any major departure from the other known teleost sequences. However, a repeat of CA dinucleotide at the 3′ UTR is unique and the number of repeats is varied in different catfishes. In *H. fossilis*, there are 19 repeats, while in *C. batrachus*, there are only 4 repeats. The putative *Ictalurus* VT precursor gene from the database do not show any CA repeat. The available sequence read ended with the coding region of the neurophysin and did not extend to the 3′ UTR region so that no CA repeat was observed. It may be inferred that the clone has CA repeats in the 3′ UTR, as it belongs to the microsatellite enriched library. It is very likely that the CA repeats may be a conserved feature of the VT precursor genes of catfishes and may have a regulatory role.

The deduced amino acid sequence of the VT precursors shows that, like all other members of neurohypophyseal nonapeptide precursor superfamily, it has a multi- domain structure, each having a separate function. Apart from the signal peptide, the vasopressin family peptides have a three domain structure. The nine amino acid hormone forms the smallest domain, the most biologically active region, and is released with a C-terminal amidation after a three-step enzymatic process. The second domain called neurophysin is joined to the hormone moiety with a GKR, which is the site of the three-step enzymatic process of cleavage of the hormone and amidation. The N-terminal 9 amino acid of the neurophysin is poorly conserved, while the rest is conserved. The third domain is copeptin which is poorly conserved except for the presence of a leucine-rich core (de Bree and Burbach, 1998). The catfish VT precursors like the vasopressin family peptides have the three domain structure. Only in mammals, a second cleavage apart from the hormone-neurophysin cleavage has been reported, which separates the copeptin from the neurophysin. The copeptin domain possesses N- linked glycosylation sites in mammals, amphibians and lungfish (Hyodo et al., 1997) but unlike in teleosts. The catfish VT copeptin domain does not have a N-linked glycosylation site nor is cleaved. The isolated IT precursor has a multi-domain structure too and, like VT/VP precursors, has an extended C-terminal with a leucine-rich core, while no other vertebrate pre-prohormone in the neutral line has got an extended C-terminal.

### Tissue expression profile of nonapeptides and functional implications

In *H. fossilis*, VT and IT genes are expressed only in the brain and ovary. In a previous study, the distribution of VT has been reported in the preoptico-hypophyseal neurosecretory system of the catfish (Singh and Joy, 2008). Brain VT showed seasonal variation associated with the annual reproductive cycle of the catfish with an increase during the recrudescent phase and a decrease after spawning. The expression of the VT and IT genes shows differences in tissue and seasonal transcript levels. The transcript levels are high in the brain than ovary. In the ovary, the expression was confined to the follicular layer since the denuded oocytes did not show any signal. In the seasonal study, the VT and IT precursor transcripts in the brain and ovary show significant variations with higher levels during the reproductive phase, suggesting a prominent influence of the reproductive factors on the transcriptional activity of the genes. The expression of both VT and IT precursor genes in the ovary points to local synthesis of the peptides for a paracrine role in ovarian activity. This confirms the earlier findings from our laboratory showing HPLC characterization of both VT and IT, and immunocytochemical localization of VT in the follicular layer (Singh and Joy, 2008). The ovarian VT levels vary with the annual ovarian cycle closely associated with recrudescence and spawning (Singh and Joy, 2008). Moreover, steroid hormones estradiol-17β and 17, 20 β- dihydroxypregnen-3-one, the maturation- inducing steroid, modulate VT secretion as in the brain (Singh and Joy, 2009a, 2011). *In vitro* studies with ovarian follicles have showed that VT stimulated steroidogenesis, oocyte final maturation, oocyte hydration, ovulation and prostaglandin secretion (Singh and Joy, 2010, 2011; Joy and Singh, 2013). Gwee et al. (2009) have shown VT expression in the ovary of elephant shark. Bobe et al. (2006) have reported VT and IT mRNAs in the preovulatory ovaries of rainbow trout, while the transcripts were not detected in the ovary during the other stages of reproduction. In mammals, there are several reports of the presence of VP and OT in nonneuronal peripheral sites, where they have a paracrine role (Wathes et al., 1983; Clements and Funder, 1986; Sernia et al., 1994; Mechaly et al., 1999). Similarly, in other classes of vertebrates like aves, presence of VT has been reported in the reproductive tissues (Saito and Grossmann, 1999). The peripheral expression of VT and its homolog signifies a phylogenetic pattern and the phenomenon seems to be conserved in all major groups of vertebrates.

### Evolutionary history of teleost NH precursors from phylogenetic analysis, genomic locations and synteny analysis

Phylogenetic inference from the present study shows that the precursor paralogs in some vertebrate groups (cartilaginous fish, coelacanth, mammals) are closer than their homologs in other groups. Similarly, studies in salmons (Hyodo et al., 1991; Suzuki et al., 1992) and flounder (Warne et al., 2000) showed that the VT and IT precursors share high sequence identity. Gwee et al. (2008, 2009) also showed that the sequence identity between the respective precursor paralogs is very high in coelacanth and elephant shark. The studies also reported that the high sequence similarity was mostly in the central portion of the neurophysin. Events of gene conversion between the paralogous genes may be responsible for diluting the distance created by the long evolutionary history and make them seem more closely related than what they actually are, typifying “concerted evolution.” Concerted evolution is common in duplicated genes, for example, as reported in rRNA genes, the highly conserved histone and ubiquitin gene families, and the heat shock protein gene family (Nei and Rooney, 2005). For the NH precursors, gene conversions seem to encompass the central neurophysin region and, therefore, the high sequence similarity in the central portion. Further evidence in favor of frequent events of gene conversion in between the nonapeptide paralogs is given for the bovine VP and OT precursor mRNA and avian NH precursors, having their central neurophysin not merely similar but identical pointing to a recent event of gene conversion (Ruppert et al., 1984; Levy et al., 1987). Gwee et al. (2008) found a low GC3 content in all the three exons of coelacanth VT and MT precursors as compared to bovine and human VP and OT genes, and attributed the sequence similarity in coelacanth to purifying selection rather than due to gene conversion. In mammals and yeast, gene conversion events are known to be GC-biased so that the GC content increases after gene conversion (Galtier, 2003; Marais, 2003; Noonan et al., 2004; Duret and Galtier, 2009). However, a recent study showed that gene conversions might not always be GC-biased (Assis and Kondrashov, 2012) and in such a scenario one of the evidence in favor of gene conversion can be incompatibility between phylogenetic tree and known duplication history (Mansai and Innan, 2010), as is the case in the present study. The neurophysin is only concerned with precursor processing and axonal transport which is common to all nonapeptides in contrast to the hormone moieties that evolved under the constraints of stringent and differential ligand- receptor selectivity in the two families. Hence, gene conversion events encompassing only the neurophysin might have occurred during evolution. This might also be facilitated by the 3-exon structure of the nonapeptide genes where the central neurophysin is encoded by a separate exon, i.e., the 2nd exon.

Based on the gene structures of salmon VT and IT, Urano and Ando (2011) had proposed a distinct and independent origin of the teleost IT as compared to the neutral hormone precursors of other vertebrates. Evidence in support of it is the presence of an extended C-terminal in IT precursors with a leucine-rich core, like in VT/VP. However, the authors have added that the molecular divergence of VT and IT in teleosts is complicated because of the 3R. The recent addition of the spotted gar sequences in the database and the analysis in the present study has prompted a re-apprisal. The presence of IT with the extended C-terminal in the spotted gar suggests an origin of IT in the actinopterygian lineage before the 3R. The spotted gar has an additional neutral peptide with a short C-terminal, which may be related directly to the evolutionary line of other vertebrate neutral hormone precursors that has originated at the base of the gnathostome lineage. In no teleost studied till date, a neutral peptide with a short C-terminal has been reported. Therefore, it may be hypothesized that the neutral hormone precursor gene with the short C-terminal might have originated very early in vertebrates, as documented in the cartilaginous fish (Hyodo et al., 2004; Gwee et al., 2009) and this early neutral hormone precursor might be the evolutionary forerunner of MT and OT. In teleosts, this neutral hormone precursor gene might have been secondarily lost or needs to be traced in the teleost lineage.

An interesting result that surfaced due to the addition of the catfish nonapeptide precursor sequences in the phylogenetic study is about the differences in the evolutionary distance shared by the precursor paralogs of different groups within teleosts. While the Acanthopterygii VT and IT precursors share a very small evolutionary distance (0.269) between them, the Ostariophysi VT and IT precursors are phylogenetically distant (0.422). The distance between the VT precursors and IT precursors of Acanthopterygii and Ostariophysi is also high (0.426 and 0.452, respectively). Phylogenetic clustering of the teleost NH precursors further supports these results. In the phylogenetic tree, the Acanthopterygii VT and IT precursors clustered together and seemed to have diverged from a common lineage. While the Ostariophysi IT precursors formed a definite cluster that included the catfish IT precursors, one of the IT precursors of the cavefish and the zebrafish IT precursor. The VT precursors of the catfish and one of the cavefish make a cluster which does not include the zebrafish VT precursor. The results from the distance calculation between the groups and the phylogenetic tree suggest that underlying the apparent homogeneity in all teleosts having VT and IT, there might be a difference in the origin of VT and IT precursors in different groups, i.e., these may be lineage-specific paralogs.

The analysis of the genomic loci of the NH hormone genes and the synteny analysis of the loci in different groups of teleosts further support the above assumption. Gwee et al. (2008, 2009) showed that in coelacanth, lamprey, and elephant shark (cartilaginous fish), the NH gene loci has a conserved synteny with the tetrapod NH loci. In fugu, there is a rearrangement. In the present study, the NH gene loci were studied in the spotted gar and teleosts belonging to different groups, which are known to have suffered independent gene losses during the process of diploidization (Garcia de la serrana et al., 2014). Our study indicates that, indeed, the loci and 3R paralogons show the footprints of rearrangements and differential losses of the NH gene paralogs in the different teleost lineages. On examination of the different genes linked with the nonapeptide genes in the spotted gar chromosome segment and the human linkage groups (as a tetrapod reference), it may be inferred that a rearrangement in the position of the genes in the nonapeptide loci of the spotted gar occurred by the fusion of the two blocks of chromosomes after the origin of IT in the actinopterygian lineage, disrupting the tandem arrangement of the genes, before the 3R. After the 3R, in teleosts the two paralogous blocks (3R paralogons) harboring the nonapeptide genes suffered further rearrangement and differential gene losses during the process of diploidization. The rearrangement was common judging from the conserved synteny in both the NH gene loci and its paralogon in all teleosts. However, the gene losses in these loci were differential in the two teleost superorders Acanthopterygii and Ostariophysi. This led to the linked and nonlinked *avp*-like and *oxt*-*like* genes in these different groups. In the acanthopterygian lineage, both the genes might have been lost from the same paralogon maintaining the linked situation of the two genes similar to the spotted gar, i.e., the pre 3R genome. In the zebrafish, *avp* like and *oxt*-like genes were lost one each from two paralogons so that a single copy of the NH hormone genes was maintained and they remained unlinked. It is difficult to reconstruct the sequence of events that can explain the present picture of synteny in the NH loci and also the inconsistent phylogenetic relation between the VT and IT precursors in the different groups of teleosts. However, it may be inferred that in the base lineage of acanthopterygians, after one pair of VT and IT precursor genes was lost from one of the 3R paralogon, an event of gene conversion might have occurred between the remaining pair. This might have occurred in the time period between the 3R and the explosive adaptive radiation and could explain the phylogentic relation of the Acanthopterygii VT and IT precursors. The Acanthopterygii VT precursors and Acanthopterygii IT precursors make two distinct clusters but share a very close phylogenetic relation. In the Ostariophysi, the loss of the NH precursor paralog was differential as compared to the Acanthopterygii with VT and IT precursor genes being lost one each from the two 3R paralogons. Rapid speciation event might have occurred after this. Gene conversion might not have occurred in the ostariophysian lineage. This explains the unlinked state of the NH paralogs in the zebrafish and also the distant relation shared by the VT and IT precursors of the Ostariophysi. The situation in the cavefish is difficult to explain. It is likely that the VT precursor gene was lost from one of the 3R paralogons while in the other both genes were retained so that a pair of linked genes remained. An independent event of gene conversion might also have occurred in between the two linked genes explaining the close phylogenetic relation between the two. The evolutionary history of the VT precursor gene of the cavefish which is unlinked can be easily traced phylogenetically as it clusters with the catfish VT precursors. However, the synteny analysis shows that it is present in a unique genomic context which shares a conserved synteny with neither the tetrapod nor the teleost NH loci, making it impossible to arrive at a possible origin of this gene. Genomic information of the NH loci from more teleost species belonging to the Ostariophysi may be helpful in explaining this. Our future direction of work would involve isolating the NH gene loci in the catfish so that the evolutionary history of the VT and IT genes in the Ostariophysi may be inferred more clearly. With the available information from the phylogeny and synteny analysis, it is clear that in the different groups of teleosts the VT and IT precursors are lineage-specific paralogs arising from differential loss of the 3R paralogs in the different superorders. The independent yet consistent retention of VT and IT in the different groups despite the catastrophes of genome duplications, diploidizations and gene conversions might have been directed by stringent ligand- receptor selectivity, established early during the vertebrate evolution.

## Conclusion

The complete VT and IT precursors were cloned from *H. fossilis*, and partially from *C. batrachus*. The deduced precursor proteins have the characteristics of the NH precursors. The precursors express in the brain and follicular envelope of the ovary. The expression in both brain and ovary shows seasonal variation with higher expression in the reproductive phases. The phylogenetic analysis of the vertebrate NH precursors and the synteny analysis of the NH gene loci show that the VT and IT precursors are lineage-specific paralogs in the superorders of Teleostei (Acanthopterygii and Ostariophysi).

### Conflict of interest statement

The authors declare that the research was conducted in the absence of any commercial or financial relationships that could be construed as a potential conflict of interest.
