# Supplementary material for: Molecular cloning, sequencing and tissue expression of vasotocin and isotocin precursor genes from Ostariophysian catfishes: phylogeny and evolutionary considerations in teleosts
Source: Front Neurosci. 2015 May 15;9:166. doi: 10.3389/fnins.2015.00166 (PMC4432659; doi:10.3389/fnins.2015.00166)
Supplement: Supplementary file 1 [file Table1.DOCX]

Supplementary table 1. Sequences from GenBank of species with their accession

numbers used for phylogenetic analysis.

| S.No. | Name | Acc. no |
| --- | --- | --- |
|  | *Danio rerio* IT | AAL50209.1 |
|  | *Takifugu rubripes* IT | AAC60289.1 |
|  | *Platichthys flesus IT* | BAA98141.1 |
|  | *Halichoeres trimaculatus* IT | ADB28876.1 |
|  | *Parajulis poecilepterus* IT | ABB90893.1 |
|  | *Sparus aurata* IT | AGO05922.1 |
|  | *Oryzias latipes* IT | NP_001265759 |
|  | *Xiphophorus maculatus* IT | XP_005794979 |
|  | *Amphiprion melanopus* IT | AEB00560 |
|  | *Astyanax mexicanus* IT (LOC103043649) | XP_007254134.1 |
|  | *Astyanax mexicanus* IT (LOC103044969) | XP_007254051.1 |
|  | *Clarias batrachus* IT | Not submitted |
|  | *Heteropneustes fossilis* IT | AFW98246.1 |
|  | *Ictalurus punctatus* IT | BE213165 |
|  | *Platichthys flesus* VT | BAA98140.1 |
|  | *Triakis scyllium* phasitocin | BAD27478.1 |
|  | *Triakis scyllium* asvatocin | BAD27477.1 |
|  | *Torpedo marmorata* isotocin | AAA78284.1 |
|  | *Latimeria menadoensis* mesotocin | ABZ04536.1 |
|  | *Protopterus annectens* mesotocin | BAG66062.1 |
|  | *Neoceratodus forsteri* Phemesotocin | BAA24027.1 |
|  | *Catostomus commersonii* VT 2 | AAA4919.1 |
|  | *Oryzias latipes* VT | BAM15897.1 |
|  | *Epinephelus coioides* VT | ADF36550.1 |
|  | *Thalassoma bifasciatum* VT | AAN87838.1 |
|  | *Parajulis poecilepterus* VT | ABB90892.1 |
|  | *Halichoeres trimaculatus* VT | ADB28877.1 |
|  | *Cyprinodon variegatus* VT | ACZ01984.1 |
|  | *Amphiprion melanopus* VT | AEB00559.1 |
|  | *Danio rerio* VT | NP_840078.1 |
|  | *Haplochromis burtoni* VT | AAM70492.1 |
|  | *Sparus aurata* VT | CCA65463.1 |
|  | *Takifugu rubripes* VT | AAC60293.1 |
|  | *Astyanax mexicanus* VT (LOC103030472) | XP_007252147 |
|  | *Astyanax mexicanus* VT (LOC103042813) | XP_007254131 |
|  | *Heteropneustes fossilis* VT | AFO70133.1 |
|  | *Ictalurus punctatus* VT | BM495247 |
|  | *Clarias batrachus* VT | Not sumitted |
|  | *Triakis scyllium* VT | BAD27476.1 |
|  | *Neoceratodus forsteri* VT | BAA24026.1 |
|  | *Protopterus annectens* VT | BAG66061.1 |
|  | *Latimeria menadoensis* VT | ABZ04535.1 |
|  | *Lethenteron camtschaticum* VT | BAA06668.1 |
|  | *Eptatretus burgeri* VT | BAA06668.1 |
|  | *Podarcis siculus* VT | AAL15166.1 |
|  | *Podarcis siculus* mesotocin | AAL15165.1 |
|  | *Taeniopygia guttata* VT | XP_002190083.1 |
|  | *Coturnix coturnix* VT | AAX18227.1 |
|  | *Gallus gallus* VT | CAA38923.1 |
|  | *Taeniopygia guttata* mesotocin | XP_002190003.1 |
|  | *Bufo japonicus* VT | AAA48556.1 |
|  | *Plethodon shermani* VT | ABP88922.1 |
|  | *Taricha granulosa* VT | ABP88920.1 |
|  | *Typhlonectes natans* VT | AAF76847.1 |
|  | *Bufo japonicus* mesotocin | AAA48555.1 |
|  | *Typhlonectes natans* mesotocin | AAF76848.1 |
|  | *Taricha granulosa* prepro-mesotocin | ABP88921.1 |
|  | *Plethodon shermani* prepro-val4 mesotocin | ABP88923.1 |
|  | *Rattus norvegicus* Oxytocin | CAA31281.1 |
|  | *Homo sapiens* Oxytocin | AAA98806.1 |
|  | *Rattus norvegicus* VP | CAA25795.2 |
|  | *Homo sapiens* VP | AAA6129.1 |
|  | *Callorhinchus milii* VT | ACN32396.1 |
|  | *Callorhinchus milii* oxytocin | ACN32397.1 |
