# Supplementary material for: Molecular cloning, sequencing and tissue expression of vasotocin and isotocin precursor genes from Ostariophysian catfishes: phylogeny and evolutionary considerations in teleosts
Source: Front Neurosci. 2015 May 15;9:166. doi: 10.3389/fnins.2015.00166 (PMC4432659; doi:10.3389/fnins.2015.00166)
Supplement: Supplementary file 2 [file Table2.DOCX]

| Species  Supplementary table 2. Details of chromosome blocks used in synteny analysis | NCBI assembly version | Genomic context | Map Position | Remark | Ensembl assembly version | Contig assembly ID | Map position | Remarks |
| --- | --- | --- | --- | --- | --- | --- | --- | --- |
| Spotted gar | LepOcu1 (GCF000242695.1) | LG2 NC_023180.1 | 414kb- 640 kb | Three NH genes present | Lepisosteus oculatus version 77.1 (LepOcu1) | LG2 | 4.9 Mb- 5.02 Mb | Three NH genes present |
| Fugu | FUGU5 (GCF_000180615.1 | NC_018910.1 | 6.43 Mb- 6.48 Mb | VT and IT genes present | Takifugu rubripes version 77.4 (FUGU4) | Scaffold_11 | 2.2 Mb- 2.3 Mb | VT and IT genes present |
| Fugu | FUGU5 (GCF_000180615.1 | Ch.6 NC_018895.1 | 841 kb- 874 kb | Paralogon of NH locus but no NH genes present | Takifugu rubripes version 77.4 (FUGU4) | Scaffold_106 | 860 Kb-881 kb | Paralogon of NH locus but no NH genes present |
| Medaka | ASM31367v1 (GCF_000313675.1) | Ch.9 NC_019867.1 | 6.91 MB-6.99 Mb | VT and IT genes present | Oryzias latipes version 77.1(MEDAKA1) | Ch9 | 6.9 Mb- 7 Mb | VT and IT genes present |
| Medaka | ASM31367v1 (GCF_000313675.1) | Ch. 10, NC_019868.1 | 24.9 Mb- 25 Mb | Paralogon of NH locus but no NH genes present | Oryzias latipes version 77.1(MEDAKA1) | Ch.10 | 25216 kb-25273 kb | Paralogon of NH locus but no NH genes present |
| Stickleback | N/A | N/A | N/A | N/A | Gasterosteus aculeatus version 77.1 (BROADS1) | Group XIII | 5.1 Mb- 5.2 Mb | Harbours the VT and IT precursor genes |
| Sticleback | N/A | N/A | N/A | N/A | Gasterosteus aculeatus version 77.1 (BROADS1) | Group XIV | 14.4 Mb- 14.6 Mb | Paralogon of the VT and IT containing linkage group but do not have VT and IT precursor genes |
| Tilapia | Orenil1.1 (GCF_000188235.2) | LG12, NC_022210.1 | 21.5 Mb-21.6 Mb | VT and IT genes present | Oreochromis niloticus version 77.1 (Orenil.0) | Scaffold GL831176.1 | 4.1 Mb- 4.2 MB | VT and IT genes present |
| Tilapia | Orenil1.1 (GCF_000188235.2) | LG7,NC_022205.1 | 34.4 Mb- 34.4 Mb | Paralogon of NH locus but no NH genes present | Oreochromis niloticus version 77.1 (Orenil.0) | Scaffold GL831141.1 | 459 kb-531 kb | Paralogon of NH locus but no NH genes present |
| Zebrafish | GRCz10 (GCF000002035.5) | Ch8 NC_007119.6 | 0.8 Mb- 1.1 Mb | VT gene present | Danio rerio version 77.8 (Zv9) | Ch.8 | 0.8 Mb- 1.3 Mb | VT gene present |
| Zebrafish | GRCz10 (GCF000002035.5) | Ch.5 NC_007119.6 | 71.2 Mb- 71.3 Mb | IT gene present | Danio rerio version 77.8 (Zv9) | Ch.5 | 75.1 Mb-75.2 Mb | IT gene present |
| Cavefish | Astyanax_mexicanus-1.0.2 (GCF_000372685.1) | NW_006749370.1 | 0.8 Mb- 1.3 Mb | VT and IT genes present | Astyanax mexicanus version 77.102 (AstMex102) | Scaffold KB882183.1 | 0.8Mb- 1.1Mb | VT and IT genes present |
| Cavefish | Astyanax_mexicanus-1.0.2 (GCF_000372685.1) | NW_006749338.1 | 64 kb-733 kb | VT gene present | Astyanax mexicanus version 77.102 (AstMex102) | Scaffold KB8822151.1 |  | VT gene present but not annotated known by blast search |
| Cavefish | Astyanax_mexicanus-1.0.2 (GCF_000372685.1) | NW_006749369.1 | 0.8 Mb- 0.9 Mb | IT gene present | Astyanax mexicanus version 77.102 (AstMex102) | Scaffold KB882182.1 | 0.8 Mb- 0.9 Mb | IT gene present |
| Human | GRch38 (GCF 000001405.26 | Ch.20 NC_000020.11 | 2.8 Mb- 3.2 Mb | VP and OT gene present | Homo sapiens version 77.38(GRCh38) | Ch. 20 | 3.0 Mb- 3.2 Mb | VP and OT gene present |
| Human | GRch38 (GCF 000001405.26 | Ch.2 NC_000002.12 | 88.0 Mb- 88.1 Mb | Conserved synteny with teleost and spotted gar NH gene locus | Homo sapiens version 77.38(GRCh38) | Ch.2 | 88.0 Mb- 88.1 Mb | Conserved synteny with teleost and spotted gar NH gene locus |
| Human | GRch38 (GCF 000001405.26 | Ch.9 NC_000009.12 | 96.2 Mb- 96.6 Mb | Conserved synteny with teleost NH gene locus | Homo sapiens version 77.38(GRCh38) | Ch.9 | 96.3 Mb- 96.5 Mb | Conserved synteny with teleost NH gene locus |
| Xenopus | Xtropicalis_V7 (GCF_000004195.2) | NW_004668232.1 | 201.4 Mb- 201.6 Mb | VP and OT genes present | Xenopus tropicalis version 77.42 | Scaffold GL172841.1 | 1.4 Mb-1.6 Mb | VP and OT genes present |
| Xenopus | Xtropicalis_V7 (GCF_000004195.2) | NW_004668232.1 | 201.4 Mb-201.6 Mb | Conserved synteny with teleost and spotted gar NH gene locus | Xenopus tropicalis version 77.42 | GL172916.1 | 584.8 Kb- 653.1 Kb | Conserved synteny with teleost and spotted gar NH gene locus |
| Xenopus | Xtropicalis_V7 (GCF_000004195.2) | NW_004668232.1 | 89.4 Mb- 89.5 Mb | Conserved synteny with teleost NH gene locus | Xtropicalis_V7 (GCF_000004195.2) | 89.4 Mb- 89.5 Mb | 2.5Mb-2.6 Mb | Conserved synteny with teleost NH gene locus |
