# Supplementary figures and images for: Molecular cloning, sequencing and tissue expression of vasotocin and isotocin precursor genes from Ostariophysian catfishes: phylogeny and evolutionary considerations in teleosts
Source: Front Neurosci. 2015 May 15;9:166. doi: 10.3389/fnins.2015.00166 (PMC4432659; doi:10.3389/fnins.2015.00166)

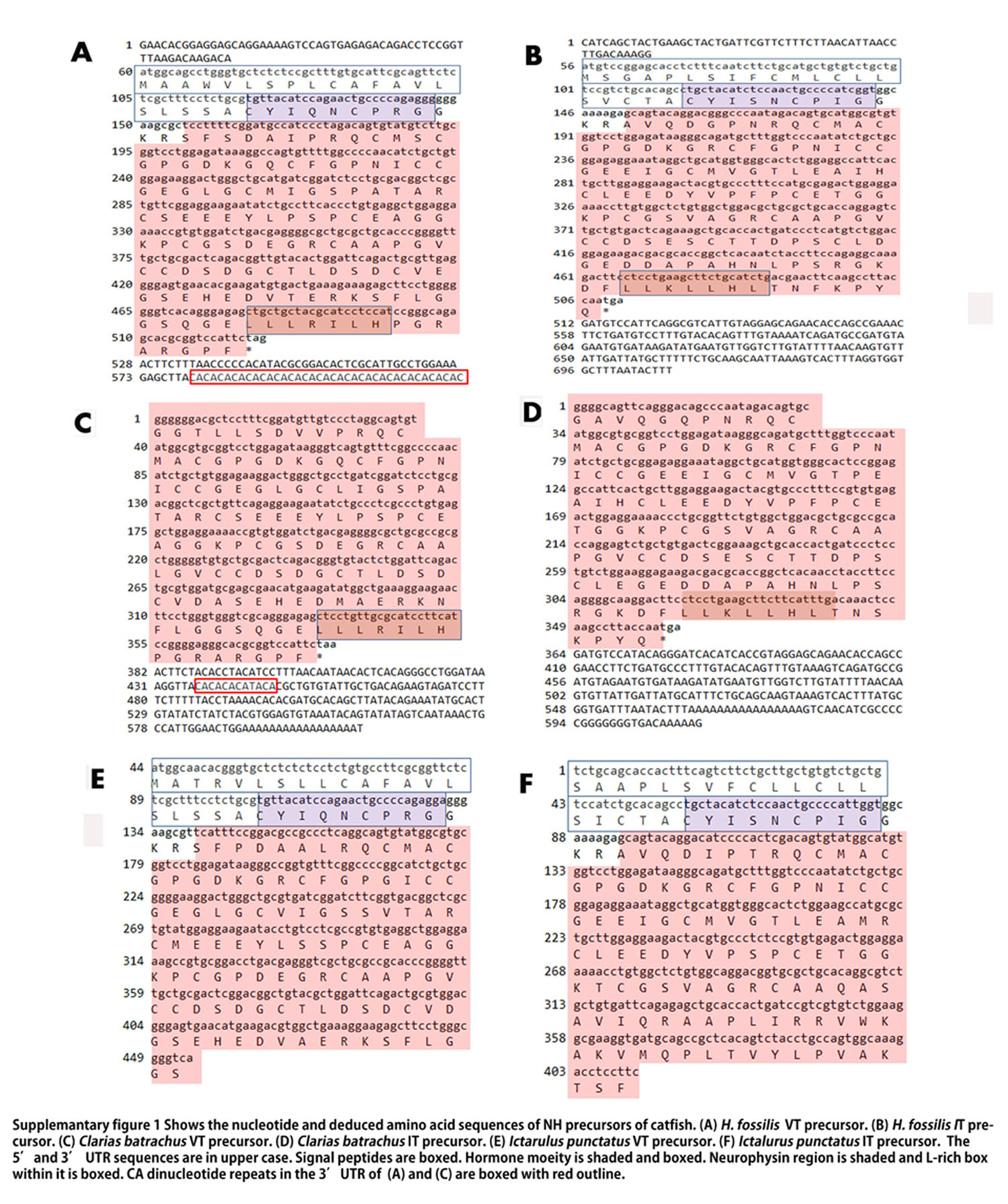

Supplement: Supplementary file 4 [file Image1.TIF]
